# Determination of Florfenicol, Thiamfenicol and Chloramfenicol at Trace Levels in Animal Feed by HPLC–MS/MS

**DOI:** 10.3390/antibiotics8020059

**Published:** 2019-05-07

**Authors:** Rosa Elvira Gavilán, Carolina Nebot, Ewelina Patyra, Beatriz Vazquez, Jose Manuel Miranda, Alberto Cepeda

**Affiliations:** 1 Department of Analytical Chemistry, Nutrition and Bromatology, Faculty of Veterinary Medicine, University of Santiago de Compostela, 27002 Lugo, Spain; rosaelviraclg@yahoo.es (R.E.G.); beatriz.vazquez@usc.es (B.V.); josemanuel.miranda@usc.es (J.M.M.); alberto.cepeda@usc.es (A.C.); 2 Department of Hygiene of Animal Feedingstuffs, National Veterinary Research Institute, 24–100 Pulawy, Poland; ewelinapatyra@gmail.com

**Keywords:** non-target feed, florfenicol, thiamfenicol, chloramfenicol, HPLC–MS/MS, validation, swine

## Abstract

Administration of florfenicol and thiamfenicol through medicated feed is permitted within the European Union, always following veterinary prescription and respecting the withdrawal periods. However, the presence of low levels of florfenicol, thiamfenicol, and chloramfenicol in non-target feed is prohibited. Since cross-contamination can occur during the production of medicated feed and according to Annex II of the European Regulation 2019/4/EC, the control of residue levels of florfenicol and thiamfenicol in non-target feed should be monitored and avoided. Based on all the above, a sensitive and reliable method using liquid chromatography tandem mass spectrometry was developed for the simultaneous detection of chloramfenicol, florfenicol, and thiamfenicol at trace levels in animal feed. Analytes were extracted from minced feed with ethyl acetate. Then, the ethyl acetate was evaporated, the residue was resuspended in Milli-Q water and the extract filtered. The method was in-house validated at carryover levels, with concentration ranging from 100 to 1000 µg/kg. The validation was conducted following the European Commission Decision 2002/657/EC and all performance characteristics were successfully satisfied. The capability of the method to detect amfenicols at lower levels than any prior perspective regulation literature guarantees its applicability in official control activities. The developed method has been applied to non-compliant feed samples with satisfactory results.

## 1. Introduction

Globalization permits food produced in one country to be sold in other countries, sometimes on an intercontinental level. However, globalization also contributes to competition between production companies. The final goal of any type of business is to have low production cost and high benefits. Low production cost is very important in food of animal origin, for which farmers fight with animal disease. The use of certain veterinary medicine is permitted to control, prevent, and treat illness. The most employed medicines, in this case, include antibiotics and antiparasitic agents. Antimicrobial medicines are sold as premixes, oral powders, oral solutions, injections, intramammary preparations pastes, oral pastes, boluses and intrauterine preparations [1]. Fenicols (chloramfenicol, thiamfenicol, and florfenicol) belong to this group of antibiotics; however, even if chloramfenicol is very effective on a broad spectrum, it is prohibited in food-producing animals within the European Union [2]. On the other hand, thiamfenicol and florfenicol can be administrated through feed, but always respecting the withdrawal periods. Regulation 2019/4/EC [3] includes these two antibiotics as an active substance for medicated feed and also in Annex II of the Regulation, indicating that cross-contamination level should be investigated during the production of medicated feed.

Techniques such as phase sorptive extraction [4], indirect competitive enzyme-linked immunosorbent assays [5], molecularly imprinted solid-phase extraction [6], high performance liquid chromatography (HPLC) with ultraviolet (UV) detection [7], capillary electrophoresis [8], and QuEChERS [9] were employed for amfenicol analysis.

The European Decision Commission 2002/657/EC states that positive samples need to be confirmed with confirmatory methods, and that HPLC–MS/MS is a good technique for confirmatory methods [10]. The use of HPLC–MS/MS has been reported for amfenicol analysis in food matrices. However, when compared with other antibiotics, such as tetracyclines, sulfonamides or penicillin, there are few methods available for amfenicols. According to the ESVAC 2015 report, these three groups of antibiotics (tetracyclines, sulfonamides, and penicillin) accounted for approximately 70% of the total sales of antibiotics in the European Union. The reported HPLC–MS/MS methods include one described by Van de Riet et al. (2013) [11] for chloramfenicol, thiamfenicol, and florfenicol in fish muscle, one developed by Barreto et al. (2016) [12] to detect the same amfenicols in poultry, swine, bovine, and fish muscle, another published by Anderson et al. (2016) [13] for florfenicol and thiamfenicol in white-tailed deer, one described for detection in milk and honey [9], and the most recent one reported for detection in egg [14].

Regarding feed samples, Pietroń et al. (2014) [15] reported an HPLC–UV method for the quantification of florfenicol and thiamfenicol in medicated feed. Later, in 2017, a similar method was reported, but the feed extract was purified by thigh layer chromatography (TLC) (Yang et al., 2017) [16]. For residue levels of amfenicols in feed, the technique most frequently employed is HPLC combined with different types of mass spectrometry. Between 30 and 300 active compounds (veterinary drugs, pesticides, and others) can be identified with low limits of detection (20 µg/kg). However, some of these methods are for screening [17,18,19,20,21] while others purify the extract with primary secondary amine (PSA) [22] or solid-phase extraction (SPE) [23].

To the best of the authors’ knowledge, no method has been found in the scientific literature that is capable of analyzing all three amfenicols (chloramfenicol, thiamfenicol, and florfenicol) at the residual levels that fulfill the requirements of the European Commission Decision 2002/657/EC. Therefore, the aim of this research article is to report a simple extraction protocol followed by HPLC–MS/MS detection of amfenicols in feed samples from different animal species.

## 2. Results

The objective of this article is to describe a simple extraction protocol and an HPLC–MS/MS method to confirm the presence of amfenicols in feed samples at trace levels.

**Figure 1:**
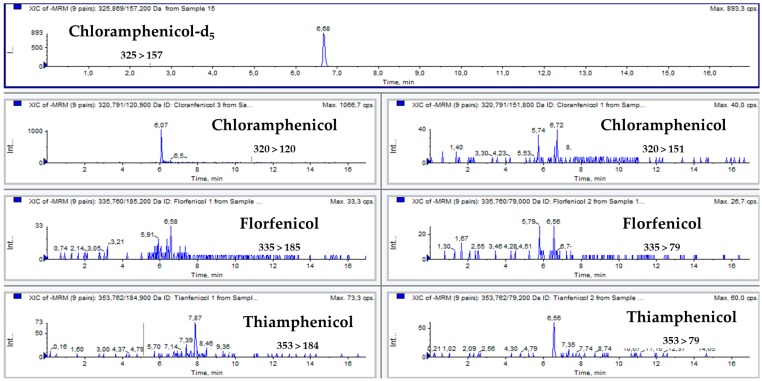
The total ion chromatogram (TIC) of a blank feed sample.

**Figure 2:**
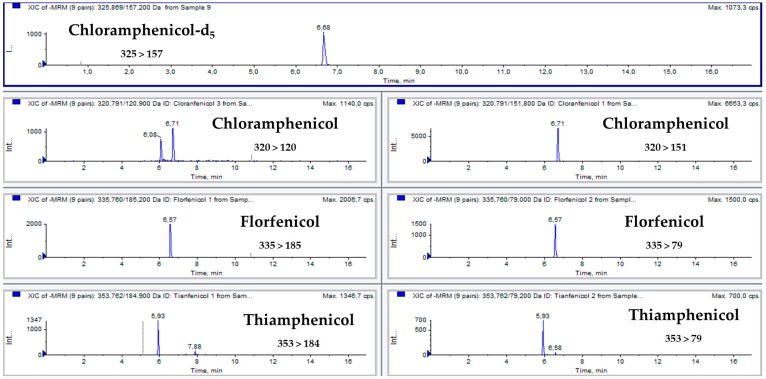
MRM chromatograms of feed samples spiked with amfenicols at the validation level of 200 µg/kg.

Figure 1 and Figure 2 show multiple reaction monitoring (MRM) transitions of the different analytes in blank feed samples and in the same blank feed samples spiked with amfenicols to a final concentration of 100 µg/kg.

### 2.1. HPLC–MS/MS Conditions

Standard solutions of 1 µg/mL of florfenicol, thiamfenicol, chloramfenicol, and chloramfenicol-d_5_ were employed to optimize their detection by the MS. The objective was to achieve a high signal intensity of representative ions through modifications in parameters such as curtain gas, ion spray voltage, source temperature, and curtain gas flow. Once precursor ions were selected for each amfenicol and optimized by manual tuning, automatic optimization was conducted (Table 1). This process permitted the evaluation of parameters such as entrance potential, collision cell potential, collision energy, and collision cell exit potential for four representative production ions. From these four ions, two were selected to conduct MRM analysis. Amfenicols were ionized by employing negative electrospray, due to the chemistry of the molecule; this ionization mode has been previously employed by other researchers in this field [14,17,18,19,20,21,22,23,24]. Acetonitrile was the first tested organic solvent as it is preferential to methanol. Acetonitrile was combined with water and buffers (ammonium acetate, ammonium formate, and ammonium hydroxide), resulting in a good intensity signal. The best resolution between peaks and signal intensity was obtained with the combination of acetonitrile and water with ammonium formate and 0.1% of formic acid.

Following the requirement of the Decision 2002/657/EC, four identification points were achieved with the use of two MRM transitions for each amfenicol and the internal standard (IS). While both MRM transitions gave identification information, quantification was implemented from the MRM that gave the higher signal to noise ratio (S/N). The ions selected for chloramfenicol and florfenicol were also employed by Cronly et al. (2010) [25] and by Shinoda et al. (2011) [26] for detection in feed matrices. Similar results occurred between the selected ion for florfenicol and the work described by Shinoda et al. (2011) and Cronly et al. (2010), though the latter reference does not include florfenicol in its method. The same ions have also employed in other type of matrices, such as wastewater [27], manure [27], poultry tissues [28], pork, porcine liver, porcine kidney, beef, bovine liver, fish, chicken [29], milk, and honey [9]. The chromatography separation was performed with various C18 columns, including LiChrospher, Symmetry Shield RP18, ZORBAX Eclipse Plus, XTerra C18, and Hypersil C18-BD. For the present work, Sunfire C18 columns were selected.

### 2.2. Extraction 

Independently of the matrix, most methods described in the scientific literature employ ethyl acetate to extract amfenicols [12,24,28,30]. Xiao et al. (2015) also employed ethyl acetate for poultry tissue but with a pressurized liquid extraction system [28]. Acetone and dichloromethane were selected by Van de Riet et al. (2013) to extract the drugs from fish muscle [11]. Acetonitrile with formic acid was employed by Boix et al. (2014) in feed samples and water combined with buffered acetonitrile by Leon et al. (2016) also for feed samples [21]. More sophisticated extraction techniques, such as QuEChERS, were employed by Liu et al. (2016) for milk and honey [9]. An immunoaffinity column was used by Luo et al. (2010) [31] for swine muscle and imprinted polymer by Ge et al. (2010) for animal tissue [6]. For the extraction of trace-level amfenicols from feed samples, 2 mL of water and 5 mL of ethyl acetate were employed with satisfactory results. However, after shaking for 20 minutes and centrifugation for the separation of the different phases, only 2 mL of the organic layer was evaporated. The evaporation of greater volumes (3, 3.5, and 4 mL) was investigated to increase the limit of detection (LOD), however, a higher matrix effect was observed, and the idea discarded.

### 2.3. Method Validation

The research group has developed, validated, and published various methods for the analysis of trace levels of veterinary drugs (coccidiostats, sulfonamides, and other antibiotics) in animal food and feed samples [32,33,34,35]. Validation for this type of sample and the matrix has typically been conducted following the European guideline (Decision 2002/657/EC) [10], which permits method implementation and comparisons in EU reference laboratories.

The Decision 2002/657/EC indicates that when no certificate reference material is available, trueness of measurements is assessed through recovery of additions of knowns amount of the analytes to a blank matrix. For amfenicols, recoveries were between −20% and +10% for the three investigated analytes (Table 2).

As with recovery, the precision under conditions of repeatability and reproducibility were evaluated using a standard addition method. Precision values were within the range provided by the European Decision; they ranged from 12% to 21% for chloramfenicol, 6.5% to 22% for thiamfenicol, and 12% to 19% for florfenicol. Currently, the presence of residue of amfenicols in non-target feed is prohibited; therefore, CCα and CCβ were calculated through employing formula for non-permitted substances. After peak identification, the area was plotted against the added concentration. CCα was the corresponding concentration at the y-intercept plus 2.33 times the standard deviation of the within-laboratory reproducibility. Similarly, for CCβ calculation, the peak area was plotted against the added concentration, and the CCα plus 1.64 times the standard deviation of the within-laboratory reproducibility of the mean measured content at the CCα equals the CCβ (2002/657/EC). While CCα values were 108, 140, and 110 µg/kg for chloramfenicol, thiamfenicol, and florfenicol, respectively, CCβ values were 116, 180, and 122 µg/kg for chloramfenicol, thiamfenicol, and florfenicol, respectively. For the selected analytes, the S/N at the CCα was around 100; values for LOD and limit of quantification (LOQ) were calculated with the feed samples spiked with amfenicols. Results indicated that the LOD and LOQ values could reach 50 µg/kg. However, to further validate the method, validation was conducted at 200 µg/kg to confirm satisfactory S/N at the CCα level of each compound and to fulfill the EU requirements.

The technique itself is very selective/specific as it uses an MRM detection mode; the use of two MRM transitions reduces the detection of other interfering peaks. Selective/specific proxies were investigated with 20 blank feed samples for different animal species. The same 20 samples were spiked at 100 µg/kg with amfenicols and other antibiotics (sulfadiazine, trimethoprim, tetracycline, and ciprofloxacin). Both the absence of interfering peaks at the Rt of the amfenicols in the two MRM transitions of each analyte and the correct identification of the analytes demonstrate the selectivity/specificity of the developed method.

### 2.4. Real Sample Collection and Analysis

Interlaboratory studies gave satisfactory results indicating the reliability and applicability of the developed method. Furthermore, the presence of the three amfenicols was investigated in 30 feed samples from feed mills. Florfenicol was the only amfenicol detected, and it was detected in one individual sample at a concentration of 0.36 mg/kg. This sample belonged to a group of four samples used to monitor florfenicol carryover after the production of a bath of medicated feed with 80 mg/kg of florfenicol. These results indicated that florfenicol carryover may occur during the manufacture of medicated feed and should be investigated in more detail. None of the samples collected from pig farms gave positive results.

## 3. Materials and Methods 

### 3.1. Chemicals, Reagents, and Stock Solutions

Florfenicol, thiamfenicol, chloramfenicol, and chloramfenicol-d_5_ (purity > 98%) were purchased from Sigma-Aldrich (St. Louis, MO, USA). Chloramfenicol-d_5_ was employed as internal standard (IS). Ethyl acetate and acetonitrile was obtained from Scharlau Chemie (Barcelona, Spain), and formic acid and ammonium acetate (purity >99% for analysis) were obtained from Acros Organics (Geel, Belgium). Purified water was prepared in-house with a Milli-Q water system from Millipore (Bedford, MA, USA). Nitrogen gas was generated using an in-house nitrogen generator from Peak Scientific Instruments, Ltd (Chicago, IL, USA).

In 20 mL volumetric flasks, 20 ± 0.01 mg of each analyte (florfenicol, thiamfenicol, chloramfenicol, and chloramfenicol-d_5_) were precisely weighed to prepare stock solutions of individual compounds in acetonitrile (to yield a final concentration of 1 mg/mL; the purity was considered when calculating specific concentrations). To prepare the intermediate stock solution (10 µg/mL), the individual solutions were mixed and diluted with acetonitrile. Each day, a working stock solution (1 µg/mL) was freshly prepared by diluting the intermediate stock solution with acetonitrile. All standards solutions were stored in the dark at −18 °C for no longer than three months.

### 3.2. Conditions for HPLC–MS/MS Analysis

The HPLC–MS/MS analyses were performed using a 1100 HPLC from Agilent Technologies (Waldbronn, Germany) attached to a QTRAP 2000™ MS from Applied Biosystems/MDS-Sciex (Toronto, Canada). The software Analyst 1.4.1 from Applied Biosystems (Toronto, Canada) was employed to control the system.

The analysis of the extracts with amfenicols was achieved with a Sunfire C18 (3.5 µm 2.1 × 150 mm) HPLC column from Waters (Milford, PA, USA), water with formic acid (185 µL), and 370 µL of ammonium formate (6.3% in water; mobile phase A), and acetonitrile (mobile phase B). Mobile phase components A and B were freshly prepared with each batch of sample. The solvents were mixed at a constant flow rate of 250 µL/min, the column temperature was maintained at 50 °C, and the injection volume was 40 µL. The gradient program was as follows: 0 min, 90% A; 1 min, 50% A; 5 min, 20% A; 7 min, 90% A; 17 min, 90% A.

The MS analysis was performed using negative electrospray ionization mode (ESI). The dwell time was 150 ms between transitions. The system uses nitrogen as nebulized gas set up at 50 psi for ion source gases 1 and 2. The source temperature was 475 °C, the ion spray voltage was −4500 V, and the curtain gas flow was 25 psi. Analytes were identified by retention time (Rt) and two multiple reaction monitoring (MRM) transitions. Table 1. compiles the MS parameters employed for the identification of each analyte.

### 3.3. Sample Extraction 

To confirm the absence of chloramfenicol, feed samples were sent to an accredited laboratory. Once the results were obtained, these samples were employed as blank feed samples to prepare matrix-matched calibration.

Quantification of the analytes was performed with matrix-matched samples spiked with the analytes at concentrations of 100, 200, 300, 400, and 1000 µg/kg. Samples were ground on a Minimoka GR-020 grinder (Lleida, Spain), and 1 g was transferred to a 15 mL Falcon conical-bottom tube. Then, 2 mL of Milli-Q water and 5 mL of ethyl acetate were added to the tube. The mixture was mixed with an IKA Minishaker MS2 (Staufen, Germany) for 20 s, shaken for 30 min at 200 rpm on a New Brunswick Scientific G25 orbital shaker (NJ, USA), and centrifuged at 7000 rpm for 10 minutes on a Kokusan H-103N centrifuge (Tokyo, Japan). Then, a volume of 2 mL was transferred to a conical glass tube and evaporated to dryness with a stream of nitrogen on a TurboVap^®^ II evaporator from Zymark (MA, USA). The dry residue was dissolved in 500 µL of Milli-Q water, filtered with a GHP Acrodisc syringe filter (0.2 µm; Waters Corporation, MA, USA), and transferred to an HPLC amber vial with an insert. The extract was stored at −20 °C until analysis, which was conducted within a day. Figure 1 shows the total ion chromatogram (TIC) of a blank feed sample, and Figure 2 shows MRM chromatograms of feed samples spiked with amfenicols at the validation level of 200 µg/kg.

### 3.4. Method Validation

Linearity range, recovery, precision (repeatability and reproducibility), selectivity/specificity, limit of detection (LOD), limit of quantification (LOQ), decision limit (CCα), and detection capability (CCβ) of the developed method were obtained, along with validation of the method. The validation was conducted following the criteria included in the Commission Decision 657/2002/EC. Blank feed samples were spiked with the amfenicols at 100, 200, 300, 400, and 1000 µg/kg for linearity verification. Calibration curves were prepared over four different days; for each day, peak area was correlated against analyte concentrations for linear regression analysis. The accuracy of the method could not be determined as certified reference materials were no available. Therefore, trueness and precision were obtained with blank feed samples spiked at 100, 200, and 300 µg/kg. Six replicates of each concentration were extracted and analyzed on the same day to obtain intra-day precision (repeatability). The same procedure was repeated over three different days for inter-day precision (reproducibility).

The selectivity/specificity was evaluated with blank feed samples spiked and non-spiked with amfenicols at the validation level (100 µg/kg). The different feed samples were provided by the manufacturers. Selectivity/specificity was evaluated with feed samples for different animal species (swine, poultry, and cattle).

### 3.5. Sample Collection and Analysis

Feed producers provided non-targeted feed samples (*n* = 30) to the laboratory. Four of these samples belonged to a feed mill and were collected after being manufactured in the same production line of medicated feed and after the cleaning batches to evaluate carryover contamination. The other feed samples were collected in pig farms and were feed employed for pig crowing. Once in the laboratory, all samples were ground and keep in plastic bottles in the dark until analysis. Interlaboratory samples from 2018 RIKILT proficiency tests were also conducted.

## 4. Conclusions

The article describes a simple a rapid confirmatory method based on HPLC–MS/MS for the simultaneous identification and quantification of residue of chloramfenicol, thiamfenicol and florfenicol in non-target feed. Since the method was validated following the EU guidelines and fulfilled the requirements of the Decision, the method can be applied by different laboratories, including reference laboratories.

## Figures and Tables

**Table 1:** Retention time (Rt), cone voltage (CV), collision energy, and precursor and product ions employed for ion identification.

Tetracycline	Rt (min)	*m*/*z* Transition	CV	Collision Energy
Chloramfenicol	6.71	320 > 151	46	26
Chloramfenicol		320 > 120	46	32
Thiamfenicol	5.93	353 > 184	56	20
Thiamfenicol		353 > 79	56	27
Florfenicol	6.57	335 > 185	61	23
Florfenicol		335 > 79	61	23
Chloramfenicol-d_5_	6.69	325 > 157	41	32

**Table 2:** Recovery (%), repeatability (CV%), reproducibility (CV%), CCα (µg/kg), CCβ (µg/kg), LOD (µg/kg) and LOQ (µg/kg) of Chloramfenicol, Tiamfenicol and Florfenicol.

	Fortification Level (µg/kg)	Recovery (*n* = 18)	Repeatability (*n* = 6)	Reproducibility (*n* = 18)	CCα	CCβ	LOD	LOQ
Chloramfenicol	100	81	21	16	108	116	25	40
	200	88	12	16				
	300	89	15	19				
	Mean	86	16	17				
Tiamfenicol	100	81.41	6.5	22.35	140	180	75	100
	200	97.69	12.58	21.61				
	300	103.83	18.97	21.98				
	Mean	94.31	12.69	21.98				
Florfenicol	100	96.61	14.64	18.77	110	122	50	75
	200	94.680	12.97	12.32				
	300	90.342	13.22	15.22				
	Mean	93.88	13.61	15.44				
